# Studying the role of *CASP8* GGGAGA repeat expansion in Alzheimer's disease

**DOI:** 10.1002/alz70855_102945

**Published:** 2025-12-23

**Authors:** Andrea Suárez, Alexandra N. Melloni, Bradley T. Hyman, Lien Nguyen

**Affiliations:** ^1^ University of Florida, Gainesville, FL, USA; ^2^ Massachusetts Alzheimer's Disease Research Center, Charlestown, MA, USA; ^3^ Massachusetts General Hospital, Boston, MA, USA; ^4^ MassGeneral Institute for Neurodegenerative Disease, Charlestown, MA, USA; ^5^ Massachusetts General Hospital, Harvard Medical School, Boston, MA, USA; ^6^ Harvard Medical School, Boston, MA, USA; ^7^ MassGeneral Institute for Neurodegenerative Disease, Massachusetts General Hospital, Harvard Medical School, Charlestown, MA, USA

## Abstract

**Background:**

Alzheimer's disease (AD) is a progressive neurodegenerative disorder and the most common form of dementia. While various genetic mutations contribute to AD risk, the full genetic landscape of the disease remains unclear. Tandem repeat expansion mutations have been implicated in a subset of neurodegenerative disorders. Recently, a repeat expansion variant in *CASP8* (*CASP8*‐GGGAGA‐AD‐R1) has been associated with an increased risk of AD with odds ratio of 2.2 (*p* = 3.1 x 10^‐5^). These results raise the question of how the *CASP8*‐GGGAGA‐AD‐R1 sequence variant contributes to AD.

**Method:**

We developed induced pluripotent stem cells (iPSC) patient derived models from AD cases with and without *CASP8*‐GGGAGA‐AD‐R1 variant and control cases to study *CASP8‐*related pathogenic pathways. We also developed CRISPR/Cas9 editing systems to excise the *CASP8*‐GGGAGA‐AD‐R1 sequence to generate isogenic cell lines. Neuronal cultures developed from parental and isogenic iPSCs will be studied for disease‐relevant molecular and pathogenic phenotypes.

**Result:**

We have generated iPSCs using fibroblast cells derived from 4 *CASP8*‐GGGAGA‐AD‐R1(+) AD, 5 *CASP8*‐GGGAGA‐C‐Var(+) AD, and 5 control cases. The iPSC lines show pluripotent markers and normal karyotypes. Repeat primed PCR (RP‐PCR) and long‐range PCR (LR‐PCR) showed the genotypes of *CASP8* GGGAGA repeats are consistent with those in fibroblasts. For CRISPR/Cas9 editing of the *CASP8* repeat expansion locus, we successfully cloned a plasmid to express Cas 9 protein, a fluorescence marker (mCherry and GFP) and guide RNA (gRNA) that target the upstream and downstream unique sequences of the *CASP8* repeat expansion locus. Editing efficiency was tested using HEK293T cells and iPSCs, with GFP and mCherry fluorescence signals detected upon 17 and 30 hours post‐transfection, demonstrating a successful expression of Cas9 proteins. LR‐PCR using genomic DNA extracted from HEK293 transfected cells show expected cut size upon transfection with Cas9 systems targeting the *CASP8* repeat loci.

**Conclusion:**

We successfully developed patient‐derived models to investigate the role of the *CASP8*‐GGGAGA‐AD‐R1 repeat expansion in AD. Through CRISPR/Cas9, we demonstrated efficient excision of the mutation in HEK293 cells. Moving forward, we will apply this system to patient models to assess whether removing the mutation can mitigate disease phenotypes, providing insights into AD mechanisms and potential therapeutic strategies.

